# Operating Room Noise Environment and Behavior in Children Undergoing General Anesthesia: A Randomized Controlled Trial

**DOI:** 10.1155/2024/4838649

**Published:** 2024-08-16

**Authors:** Marc Bozych, Nguyen K. Tram, Julie Rice-Weimer, Richard S. Cartabuke, Joseph D. Tobias, Jamie Huffman, Christian Mpody, Joshua C. Uffman

**Affiliations:** ^1^ Department of Anesthesiology & Pain Medicine Nationwide Children's Hospital, Columbus, OH, USA; ^2^ Department of Anesthesiology & Pain Medicine The Ohio State University, Columbus, OH, USA

## Abstract

**Background:**

Excessive operating room noise impairs communication, distracts from monitoring equipment, and may increase patient and provider stress.

**Aim:**

This study investigates the effects of reduced noise on perioperative behavior in children undergoing general anesthesia and on anesthesia provider response time.

**Methods:**

Healthy children (the American Society of Anesthesiologists class I-II), 2–8 years of age, and their anesthesia providers were randomized into a control or treatment group exposed to reduced stimulation during induction and emergence. Primary outcomes were patient behavior and provider response time. Secondary outcomes were postoperative pain scores, provider responses exceeding 30 seconds, and median and maximum noise exposure.

**Results:**

64 children (27 females and 37 males) were randomized into a control or treatment group, of whom 32 (50%) underwent tonsillectomy/adenoidectomy and 32 (50%) underwent dental procedures. The average age was 4.6 (SD 1.43) years. Children exposed to reduced noise were less likely to be “fussy about eating” (*p*=0.042), more “interested in what goes on around them” (*p*=0.008), and had fewer temper tantrums (*p*=0.004) on postoperative day one or two and on postoperative day five, six, or seven. No other differences were found between groups in behavioral assessment scores or provider response times.

**Conclusions:**

Our study is the first to show that a low-stimulus environment improves postdischarge behavior. Provider response time was unaffected by reduced noise, and the average and peak noise exposure levels did not exceed national safety guidelines. This trial is registered with NCT03507855 and NCT03504553.

## 1. Introduction

Intraoperative adverse events may represent up to 65% of all adverse events in the hospital setting, with the majority of adverse surgical events being attributed to preventable human factors [1]. One of the major human factors contributing to adverse events is miscommunication, often as the result of excessive noise in the operating room (OR) [2]. The World Health Organization recommends that ambient noise in the hospital should not exceed 35 dB during the day [3]. However, a review of noise levels in the OR found an average of 51 to 75 dB [4]. At these volumes, medical personnel would have to speak at 95 dB (the same level produced by an MRI machine) to be understood [5].

Healthcare providers are also adversely affected by noise in the OR. More than 95% of surveyed OR staff reported negative physiological and psychological effects (i.e., agitation, inattentiveness, anxiety, fatigue, and headaches) due to excess OR noise [6]. Furthermore, stress due to acute noise exposure has been demonstrated to impair cognitive function and short-term memory among medical professionals [7, 8]. Noise-related fatigue and worsened state of mind may further amplify mechanical or communication errors, compounding preventable adverse events. Repeated requests in the OR were five times more likely to occur during cases playing loud music than in those not playing music [9], and high noise level in the OR has even been linked to surgical site infection [10].

A loud OR environment can distress patients, distract providers, and impair communication [2, 7]. Previous studies by Kain et al. have shown decreased preoperative anxiety in patients exposed to low-stimulation on induction but did not reveal any posthospitalization changes [11]. This group also found midazolam to be superior to parental presence in reducing preoperative anxiolysis and superior (in combination with acetaminophen) at reducing negative postoperative behaviors, as compared to acetaminophen alone, while Kain et al. and Sola et al. failed to distinguish between midazolam and tablet devices [12–14]. What has not yet been shown in the literature is whether a combination of reduced noise and chemical anxiolysis can have an impact on the posthospitalization course of pediatric patients.

Induction and emergence, the most critical periods of an anesthetic, are often associated with the highest noise levels during a surgical case [4]. Our primary hypotheses were that a reduced noise environment during both induction and emergence, combined with midazolam premedication, would improve pre- and postoperative patient behavior and decrease the response time of anesthesia providers to auditory alarms.

## 2. Methods

Institutional Review Board (IRB) approval was obtained for the study at Nationwide Children's Hospital (IRB18-00203 and IRB18-00227), informed consent was obtained from patient guardians and providers prior to enrolling all participants, and the study was registered with clinicaltrials.gov (NCT03507855 and NCT03504553). The study involved two arms: one exploring the behavioral effects of a reduced-noise environment on pediatric patients undergoing general anesthesia and the second examining the reaction times of anesthesia providers working in this environment. The two arms initially began as separate studies but were then combined due to the interrelated nature of the subject matter. The primary outcome of the study was the anxiety level at induction, as measured using the modified Yale Preoperative Anxiety Scale (m-YPAS). A prior study looking at low stimulation in the OR had shown a decrease from 60 to 42 points on this metric [11]. Assuming a standard deviation of 8 points, we determined that an independent *t*-test would have 90% power to appreciate this difference with 95% confidence, provided that a minimum of 12 patients were randomized into each group. Other outcomes were compared across the two groups using unpaired *t*-tests, rank-sum tests, Chi-square tests, or Fisher's exact tests, as indicated by the event rate and distribution of the endpoints. Analysis of ordinal measures of anxiety, compliance, and posthospitalization behavior was performed via Spearman correlation coefficients, and two-tailed *p* values less than 0.05 were considered statistically significant. We therefore determined that a sample size of at least 29 pairs was needed.

### 2.1. Study Population

Patients 2–8 years of age, evaluated to be the American Society of Anesthesiologists (ASA) class I-II, undergoing tonsillectomy with or without adenoidectomy, tympanomastoidectomy, or single general abdominal laparoscopic or dental restoration procedure lasting at least 30 minutes were eligible for participation. Exclusion criteria included previous enrollment in the study, use of any antidepressant, anxiolytic, or pain medication other than acetaminophen or nonsteroidal anti-inflammatory drugs (NSAIDs), allergy to or parent refusal of midazolam, history of emergence delirium, cardiac disease other than functional heart murmur, developmental delay, and hearing loss. All children included in this study received premedication with midazolam (0.25–0.55 mg/kg to a maximum of 15 mg). Eligible staff included anesthesia providers (certified registered nurse anesthetists, pediatric anesthesiology fellows, or attending anesthesiologists) providing general anesthesia for patients who met inclusion criteria, unless they had been previously enrolled in the study.

### 2.2. Interventions

The children were randomized into one of two groups: the control group under typical OR conditions and the experimental group with reduced OR personnel, low ambient light, muted communication devices, and soft background music during the induction of and emergence from anesthesia (Bach's Air on the G String). A blocked randomization list was generated online at https://www.sealedenvelope.com using block sizes of two and four. The providers were not specifically randomized or blinded but instead followed whichever group to which their patient had been assigned. No losses or exclusions occurred following randomization. Research staff generated the random allocation sequence, enrolled participants, and assigned the participants to interventions. Patient enrollment began on October 26th, 2018, and follow-up concluded on November 29th, 2021. The trial ended when an adequate number of participants had completed the study in order to appropriately power the analysis. Only the anesthesia providers interacted with the patient during induction. No parents or caregivers were present during induction. A microphone secured near the patient's head or torso on the operating room table or to an IV pole at the head of the bed measured the decibel level continuously from the time the patient entered the OR until they were taken to the recovery room. Potential differences in results based on microphone location were not specifically evaluated. Local anesthetic was utilized by the surgeon or dentist for five of the patients from each group.

A blinded observer, using the m-YPAS instrument, evaluated each child before receiving premedication with oral midazolam, and again during induction 15–45 minutes later. Behavior during induction was also assessed using the Induction Compliance Checklist (ICC) instrument. Anesthetic management other than premedication was determined by the attending anesthesiologist, and specific data regarding management choices for induction and maintenance of or emergence from anesthesia and depth of anesthesia throughout each procedure were not recorded. Postoperatively, patient behavior was assessed by a blinded observer for pain and delirium using the Face, Legs, Activity, Cry, and Consolability (FLACC) and Pediatric Anesthesia Emergence Delirium (PAED) scales, respectively, every 10 minutes while the patient was in phase I recovery. Frequency and quantity of analgesic medications administered during phase I recovery were also recorded. All patients in the study recovered in one of two postanesthesia care rooms, where noise levels were not recorded or controlled for. In order to capture potential behavioral changes previously demonstrated to occur on postoperative day two, blinded patient guardians were asked to assess patient behavior using the modified Posthospitalization Behavior Questionnaire (PHBQ) instrument on postoperative day one or two, and again on postoperative day five, six, or seven (a total of two times for each patient). We did not record which parent or guardian made these separate assessments.

During each case, at a single randomized time coinciding with induction, an audio stimulus meant to simulate a patient monitor alarm would emit from a device secured next to the real monitors. The device emitted a sound 80–90 dB at a range of 0.5 meters. The response time of anesthesia providers to silence this alarm was recorded in each instance.

### 2.3. Data Analysis

Continuous data were presented as means with standard deviations or medians with interquartile ranges. Categorical variables were presented as frequencies and percentages. The Mann–Whitney test was used to compare the reaction time, noise level, ICC score, and m-YPAS score. The Wilcoxon test was used to compare the reaction time of providers who had participated in both the control and reduced noise groups. The repeated measures' analysis of variance (RM-ANOVA) test was used to compare the PAED, FLACC, and PHBQ scores. All statistical analysis was performed using GraphPad Prism 9.0.0 (GraphPad Software, San Diego, CA).

## 3. Results

Characteristics of study participants: the unblinded anesthesia provider cohort included 36 anesthesia providers (34 nurse anesthetists, one pediatric anesthesiology fellow, and one anesthesiologist), administering general anesthesia for patients undergoing tonsillectomy/adenoidectomy (*n* = 32 and duration 22–60 min) and dental procedures (*n* = 32 and duration 16–105 min). Although the study protocol allowed a greater variety of procedures, these case types were ultimately the most accessible due to the availability of the investigative staff. Twelve of the providers participated in both the control and reduced noise groups, the remaining 24 participated in only one group or the other. The patient study cohort included 64 patients, 10 (32.3%) females and 21 (67.7%) males in the control group and 17 (51.5%) females and 16 (48.5%) males in the reduced noise group. Differences in patient sex were not statistically significant (*p*=0.119 using Chi-square test). Patients in the control group were 2.4–7.8 years, and those in the reduced noise group were 2.2–7.9 years (Table 1). All patients, but one in the control group and all patients in the reduced-noise group, spoke English as their first language. There were no statistically significant differences between participants in the two groups.

Noise level and patient behavior: the median noise level in the OR was significantly higher in the control group, as compared to the reduced noise group (59 versus 56 dB, respectively, *p*=0.008; Supplemental Figure 1). However, the maximum noise level measured was not significantly different between the two groups (90 versus 89 dB, respectively, *p*=0.556). The provider response time to simulated audio monitor alarms was shown to be nonsignificant (*p*=0.421 for the unpaired test including all providers, *p*=0.092 for the paired test including only those providers who participated in both the control and reduced noise groups; Supplemental Figure 2). Twelve of the 36 providers were included in the paired analysis (Table 2).

**Figure 1:**
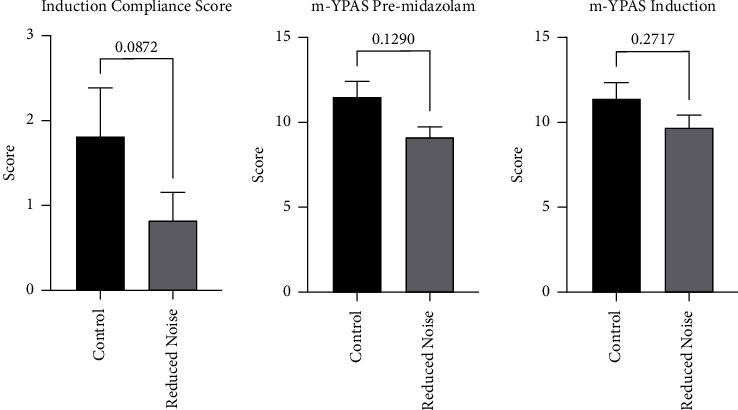
ICC and MYPAS results. ICC score represents the number of negative behaviors displayed by patients during induction (up to a maximum of 10). m-YPAS scores represent a weighted amalgam of five categories of behavior: activity, vocalizations, expressivity, arousal, and involvement of parents, with higher numbers associated with greater anxiety.

**Figure 2:**
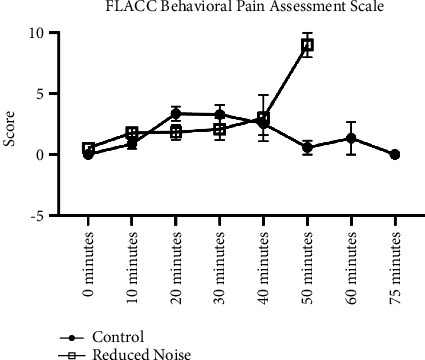
FLACC results. Each of the five metrics of the scale is scored from zero to two, for an overall rating of zero representing no expected pain to ten representing likely excruciating pain. Data for the reduced noise group were not recorded at the 60- and 75-minute marks due to patient discharge from phase I recovery.

**Figure 3:**
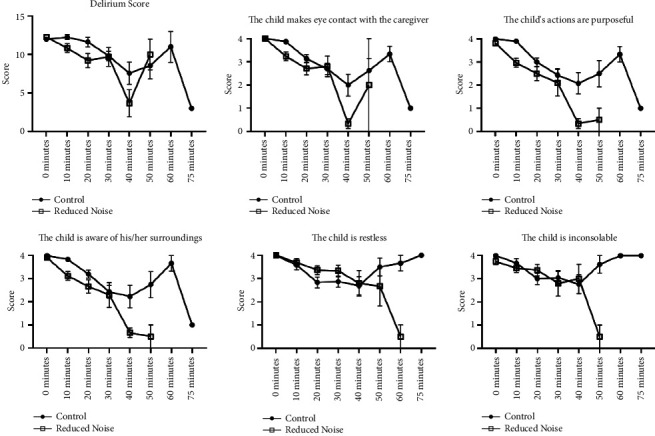
PAED scale results. Individual scores are graded subjectively from zero to four, with a higher score being associated with greater degree of delirium. Data for the reduced noise group were not recorded at the 60- and 75-minute marks due to patient discharge from phase I recovery.

**Figure 4:**
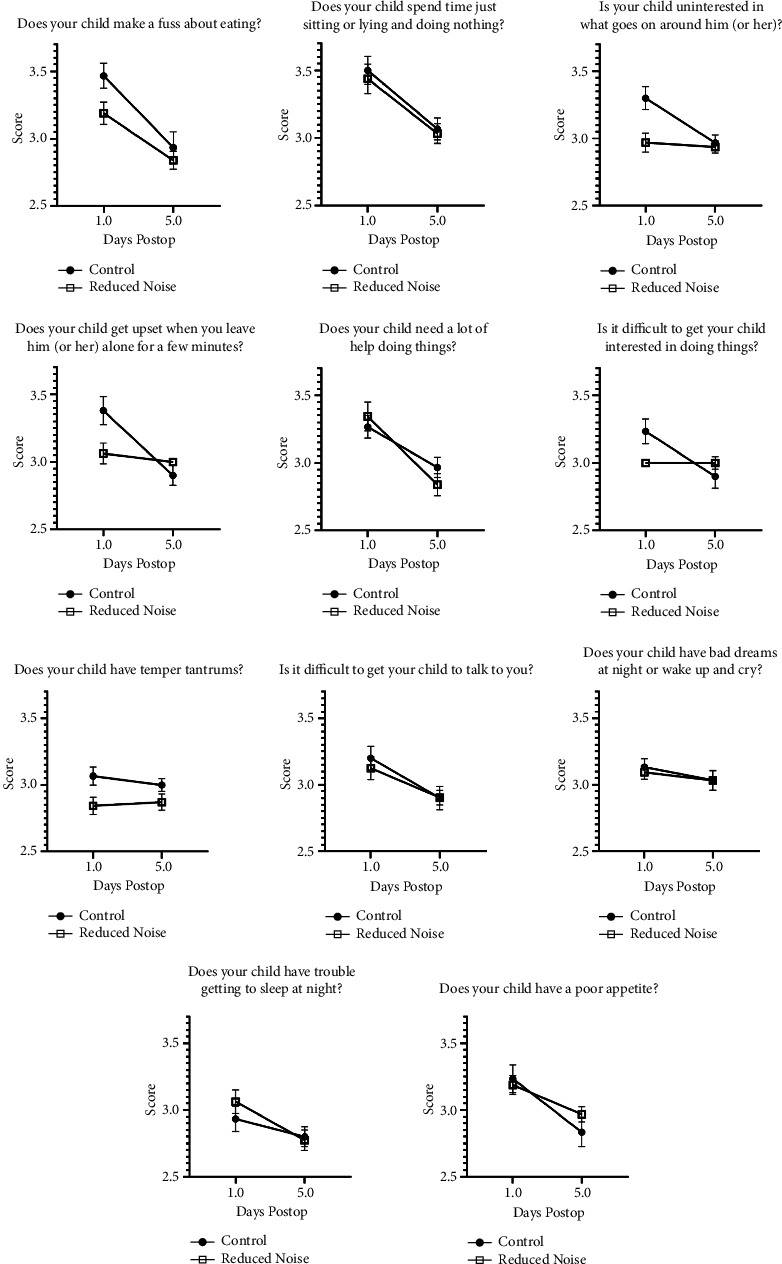
PHBQ results. Scale 1: much less than before, scale 2: less than before, scale 3: same as before, scale 4: more than before, and scale 5: much more than before.

The ICC, m-YPAS, FLACC, and PAED instruments showed no statistically significant difference in behaviors between children in the reduced noise and control groups during the preinduction, induction, and emergence periods (*p*=0.087, 0.272, 0.856, and 0.366, respectively; Figures 1, 2, and 3). Assessments for FLACC and PAED were discontinued once patients left phase I recovery. The discharge criteria at our institution are uniform across all locations where anesthesia is provided and include physiologic metrics, as well as a minimum time duration for all patients receiving intravenous opiates, nebulized epinephrine, or medication reversal (such as naloxone), as well as for patients for whom the endotracheal tube is removed in the recovery room. Shorter recovery times were incidentally observed for patients in the reduced noise group, although this was not a variable included in our official study protocol, and it was not analyzed statistically. The PHBQ assessment, however, did demonstrate significant improvements for patients regarding fussiness about eating, interest in things happening around them, and frequency of temper tantrums, as assessed on postoperative day one or two, and again on postoperative day five, six, or seven (*p*=0.042, 0.008, and 0.004, respectively; Figure 4). Patients in the reduced noise group also required, on average, fewer doses of pain medication and lower total quantity of pain medication in phase I recovery, as compared to those in the control group (Table 1).

## 4. Discussion

Young children undergoing adenotonsillectomy or dental restoration had improved postdischarge behaviors when exposed to a reduced noise environment in addition to preoperative oral midazolam anxiolysis. The reduced noise environment did not influence the response time to a simulated monitor alarm of the anesthesia providers caring for these patients during the induction of anesthesia.

Reducing preoperative anxiety and improving the quality of induction for children are large components of care for a pediatric anesthesiologist. Kain et al. showed a decrease in preoperative anxiety for patients exposed to low-stimulation conditions during induction without other preinduction anxiolytic measures (e.g., midazolam or parental presence at induction) [11], after previously showing premedication with midazolam to be superior to parental presence in alleviating preoperative anxiety [12]. Sola et al. and Marechal et al. failed to show a difference in anxiolysis between premedication with midazolam and tablet devices [13, 14]. Our results indicate that there is no further benefit in reducing anxiety during the induction of anesthesia by adding a low-stimulus environment provided that preoperative midazolam is administered. While we did not test the benefits of distraction with a tablet device or the effects of other anxiolytic medications, given their comparable benefits to oral midazolam at reducing preoperative anxiety in previous studies, we would not anticipate any further benefit from a low-stimulation environment as well.

Emergence agitation (EA) is a behavioral disturbance during the recovery from anesthesia, consisting of hallucinations, delusions, and involuntary physical activity, which is commonly seen in young children. The magnitude and severity of EA can be measured by a validated scoring system, such as the PAED [15]. Factors linked to an increased risk of EA include age, preoperative anxiety, pain, patient personality, surgical procedure, and anesthetic type. Perioperative administration of midazolam, alpha-2 agonists, opioids, ketamine, and nonsteroidal anti-inflammatory medications has been shown to decrease the incidence of EA [16]. In our study, there was no benefit to a low-stimulation environment on pain scores or the incidence of EA.

The original PHBQ, developed in 1966, was modified and revalidated, showing that children are most likely to demonstrate behavioral changes on postoperative day two [17]. In our study, postoperative behavior in children exposed to a reduced stimulation operating room environment was better on postoperative day one or two, and on postoperative day five, six, or seven, despite no differences in preoperative anxiety, postoperative pain, or EA. Since the caregivers for our patient participants were contacted on postoperative day one or two, and again on postoperative day five, six, or seven, it follows that our screening is likely to have captured behavioral changes exhibited by the children. Our findings differed from previous works that had shown a correlation between posthospitalization behavioral changes and preoperative anxiety [17], but no benefit for a low-stimulation environment when midazolam was not administered [11]. Given that we did not see a difference between the groups for either pre-operative anxiety or EA, the reason why the addition of a low-stimulation environment resulted in a reduction in maladaptive behaviors is unclear and warrants a larger study with more tightly controlled environments.

The hypothesis that the response time to a simulated audio alarm in the OR would be significantly decreased by reducing the ambient noise level was not confirmed. Despite a 2 dB reduction in the median noise level, which represents a 77% decrease in sound intensity, provider response time was not altered. Although adverse patient events and communication errors were not specifically tracked in our study, the previous work by Crockett et al. suggests that reductions in those occurrences might be achieved through significant noise reduction during critical moments of the anesthetic [2]. Survey results from that initiative determined that, at a minimum, there was a subjective belief held by anesthesia providers that noise played a significant role in OR distractions.

Median and peak noise levels did not exceed the National Institute for Occupational Safety and Health (NIOSH) guidelines, which call for no more than eight hours of noise exposure at 85 dB or four hours of exposure at 88 dB [18]. Neither of these levels was achieved during our study when total exposure time was calculated. Katz described that an upper limit of 55 dB would be needed in order to provide “universally safe conditions” in the workplace [19]. Our reduced noise group achieved a median level of 56 dB, which unfortunately still exceeds that threshold. Therefore, while our anesthesia providers were not at significant risk for long-term hearing loss due to noise exposure in the OR, there was still a potential for other adverse outcomes.

Our study had a number of limitations. All patients in our study underwent surgery of the head (i.e., in close proximity to the auditory system), which could have introduced confounding effects into our results. It would be interesting in future studies to include a greater variety of procedure types to further investigate those effects. Similarly, we included in our protocol children undergoing tympanomastoidectomy, which is a procedure often performed on individuals with underlying auditory pathology. Even though hearing loss was one of our specific exclusion criteria, and no enrolled study participants actually underwent this procedure, it may have been more appropriate to exclude this procedure from our protocol entirely. Although we noted a statistically significant difference in the noise levels between the two study cohorts, the causes of the increased noise (e.g., conversations away from the operating room table or activities such as opening equipment and OR preparation) were not investigated and identified. In addition, the noise levels in the recovery room were not measured or controlled for. It is possible that statistically significant differences existed between the two groups, adding a confounding variable. The caregivers for our patients were contacted for PHBQ assessments on postoperative day one or two, and again on postoperative day five, six, or seven, but it is possible that behavioral changes in the days between may have been notable. In addition, we did not record which caregiver was contacted on each occasion, thereby potentially introducing another confounding variable if a different observer reported on each day. For the purposes of this study, we assumed that the provider reaction time to a simulated patient monitor alarm correlated to the reaction time to a genuine monitor alarm, indicative of a precursor or in-process patient critical event. Since it was not possible to blind the providers, this may reflect, in part, the Hawthorne effect, whereby individuals increase their performance when they are aware that they are subjects in an experiment. In our study, while not significant, we saw an inversely proportional trend in reaction speed to silencing a simulated alarm with years of experience. This may in fact reflect the enhanced ability of a more experienced provider to tune out unimportant (albeit intrusive) stimuli, such as a fake alarm, while focusing on their patient during critical points of the anesthetic. For this reason, if we had succeeded in establishing statistically significant differences in response times to this alarm, it still might not have elucidated the answer to the question we ultimately sought: does a quieter OR provide a safer anesthetic environment for our patients? Future studies should focus on whether noise reduction affects more complex interactions, such as communication between members of the anesthesia team or between the anesthesia and surgical teams.

In conclusion, we have shown a benefit in postoperative maladaptive behavior in young children exposed to a low-stimulation environment, in combination with oral midazolam. Additional studies are needed to further delineate the various potential effects of a reduced noise environment for both pediatric patients and their anesthesia providers during the critical moments of induction and emergence.

## Figures and Tables

**Table 1:** Provider and patient demographics and premedication dosing.

Characteristics	Control group	Reduced noise group
Provider female sex, *n* (%)	21 (87.5%)	19 (79.2%)
Provider first language English, *n* (%)	24 (100%)	22 (91.7%)
Provider job title: CRNA, *n* (%)	24 (100%)	22 (91.7%)
Provider experience (years)	5.5 ± 0.9	5.9 ± 0.9
Patient female sex, *n* (%)	10 (32.3%)	17 (51.5%)
Patient age (years)	5.11 ± 2.68	5.07 ± 2.86
Patient first language English, *n* (%)	30 (96.8%)	33 (100%)
Patient ASA class I, *n* (%)	13 (41.9%)	13 (39.4%)
Oral midazolam dose (mg/kg)	0.40 ± 0.15	0.39 ± 0.12
Tonsillectomy/adenoidectomy, *n* (%)	15 (46.9%)	17 (53.1%)
Tonsillectomy/adenoidectomy duration (min)	36.5 ± 14.5	41 ± 19
Dental procedures, *n* (%)	16 (50%)	16 (50%)
Dental procedure duration (min)	60.5 ± 44.5	67.5 ± 27.5
Local anesthetic administered, *n* (%)	5 (16.1%)	5 (15.2%)
Mean phase one recovery fentanyl doses (n)	0.23	0.06
Mean total phase one recovery fentanyl given (mcg)	2.27	0.61

**Table 2:** OR noise levels and provider reaction times.

Characteristics	Control group	Reduced noise group	*P* value
Maximum noise (dB)	89.71 ± 1.207	88.82 ± 0.9147	0.556
Median noise (dB)	58.53 ± 0.7705	56.08 ± 0.4889	0.008
Reaction time (sec)	4.111 ± 0.6791	3.085 ± 0.3228	0.422
Reaction time, paired (sec)^∗^	4.415 ± 0.6965	3.058 ± 0.3874	0.092

^∗^Only the 12 providers who participated in both control and reduced noise groups were included.

## Data Availability

The data used to support the findings of this study are available upon request. Please contact Dr. Joseph Tobias for details.

## Additional Points

*Clinical Implications*. What is already known? Patients exposed to low stimulation on induction experience decreased preoperative anxiety, but no posthospitalization effects. Midazolam is superior to parental presence (but not superior to tablet devices) in reducing preoperative anxiety. What does this article add? Patients given midazolam premedication followed by low stimulation on induction experience posthospitalization behavioral improvement, in addition to decreased preoperative anxiety. This trial is registered with Clinicaltrials.gov (NCT03507855 and NCT03504553).

## Supplementary Materials

Supplementary Materials Supplementary Figure 1: cumulative level of noise exposure. *N* = 24 median, 24 maximum. Supplementary Figure 2: reaction time to simulated alarm. *N* = 24 unpaired, 12 paired. CONSORTCHECKLIST and CONSORT flow diagram.
