# Supplementary material for: Operating Room Noise Environment and Behavior in Children Undergoing General Anesthesia: A Randomized Controlled Trial
Source: Anesthesiol Res Pract. 2024 Aug 16;2024:4838649. doi: 10.1155/2024/4838649 (PMC11343623; doi:10.1155/2024/4838649)
Supplement: Supplementary Materials — Supplementary Figure 1: cumulative level of noise exposure. N = 24 median, 24 maximum. Supplementary Figure 2: reaction time to simulated alarm. N = 24 unpaired, 12 paired. CONSORTCHECKLIST and CONSORT flow diagram. [file 4838649.f1.zip › Noise_study_SUPPLEMENTAL_FIGURES mm.docx]

**SUPPLEMENTAL FIGURES**


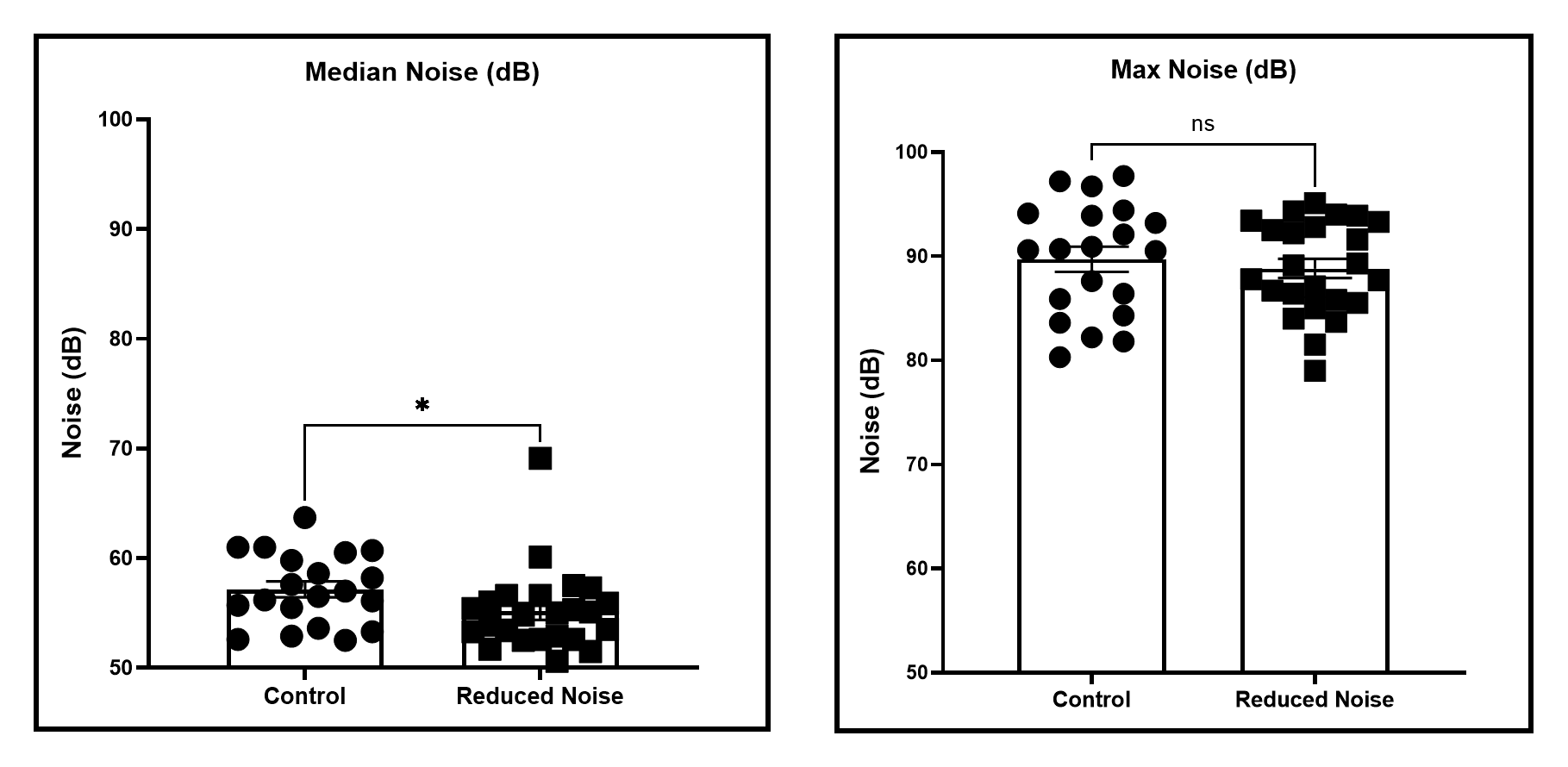


**Supplementary Figure 1.** Cumulative level of noise exposure. N = 24 median, 24 maximum.


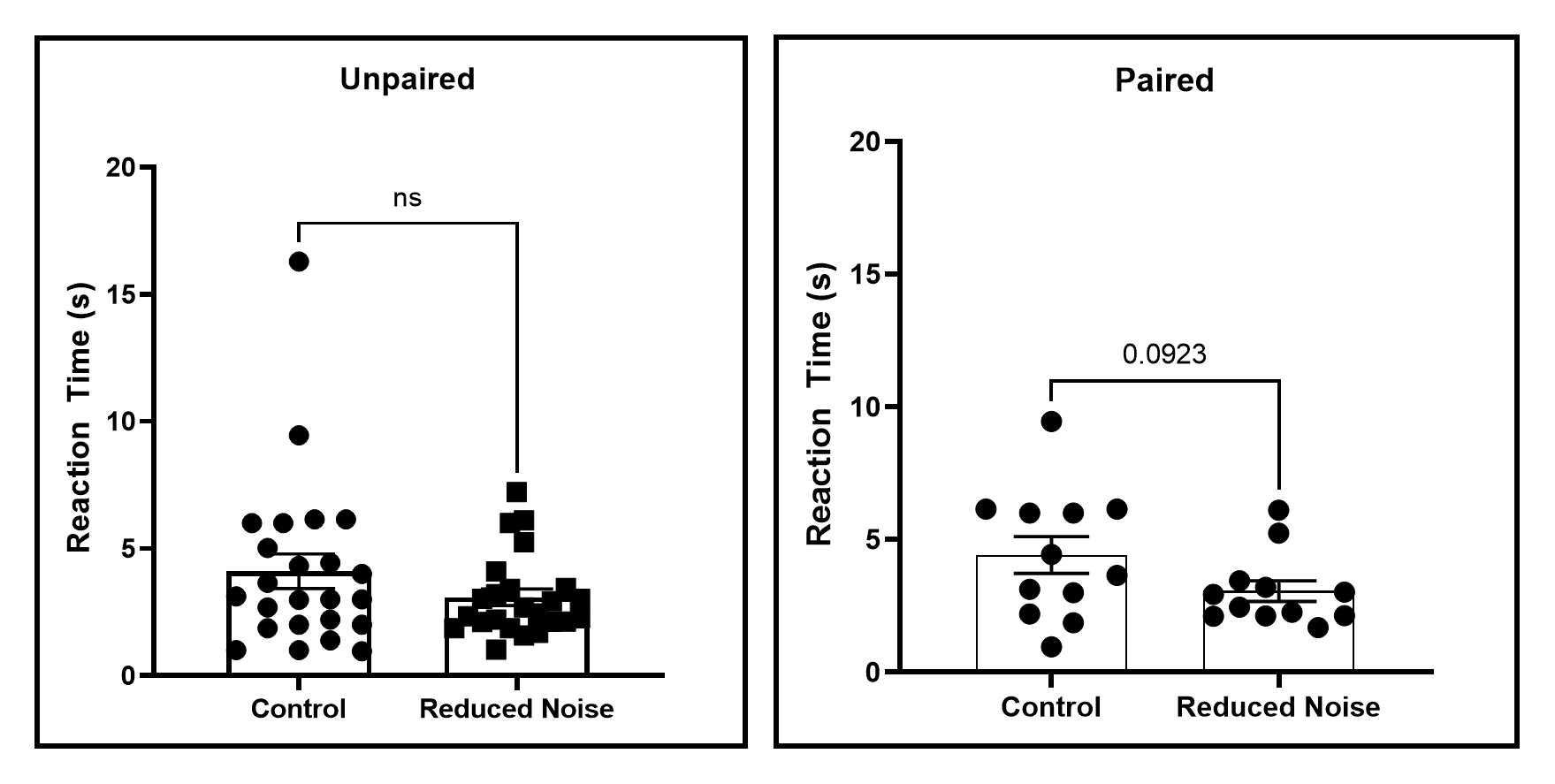


**Supplementary Figure 2.** Reaction time to simulated alarm. N = 24 unpaired, 12 paired.
